# Charged/Polar-Residue Scanning of the Hydrophobic Face of Transmembrane Domain 9 of the Yeast Glutathione Transporter, Hgt1p, Reveals a Conformationally Critical Region for Substrate Transport

**DOI:** 10.1534/g3.115.017079

**Published:** 2015-03-16

**Authors:** Anil Thakur, Anand K. Bachhawat

**Affiliations:** * Institute of Microbial Technology (IMTECH), Sector 39-A Chandigarh 160036, India; † Indian Institute of Science Education & Research (IISER) Mohali, Knowledge City, S.A.S. Nagar, Punjab 140306, India

**Keywords:** glutathione transporter, oligopeptide transporter family, charged residue scanning, TMDs, hydrophobic face

## Abstract

Unraveling the mechanistic workings of membrane transporters has remained a challenging task. We describe a novel strategy that involves subjecting the residues of the hydrophobic face of a transmembrane helix to a charged/polar scanning mutagenesis. TMD9 of the yeast glutathione transporter, Hgt1p, has been identified as being important in substrate binding, and two residues, F523 and Q526, are expected to line the substrate translocation channel while the other face is hydrophobic. The hydrophobic face of TMD9 helix consists of residues A509, V513, L517, L520, I524, and I528, and these were mutated to lysine, glutamine, and glutamic acid. Among the 16 charged mutants created, six were nonfunctional, revealing a surprising tolerance of charged residues in the hydrophobic part of TM helices. Furthermore, the only position that did not tolerate any charged residue was I524, proximal to the substrate binding residues. However, P525, also proximal to the substrate binding residues, did tolerate charged/polar residues, suggesting that mere proximity to the substrate binding residues was not the only factor. The I524K/E/Q mutants expressed well and localized correctly despite lacking any glutathione uptake capability. Isolation of suppressors for all nonfunctional mutants yielded second-site suppressors only for I524K and I524Q, and suppressors for these mutations appeared at G202K/I and G202K/Q, respectively. G202 is in the hydrophilic loop between TMD3 and TMD4. The results suggest that I524 in the hydrophobic face interacts with this region and is also in a conformationally critical region for substrate translocation.

Despite the significant progress in structure determination of membrane transporters, elucidating the dynamics of substrate movement through a transporter or a channel has remained a challenge (Elvington and Maduke 2008; Law *et al.* 2008). Crystal structures provide a static snapshot of the membrane transporter, but the substantial conformational changes that occur during substrate movement are far more difficult to capture. This latter aspect requires additional biochemical, genetic, or spectroscopic inputs to provide insights into the nature of the conformational changes (Elvington and Maduke 2008).

Over the years, a number of genetic and biochemical techniques have been exploited to obtain insights into the mechanistic workings of membrane transporters. These approaches have provided key support for our understanding, such as in the "alternating access model" of substrate transport seen in LacY permease and other transporters (Abramson *et al.* 2003; Guan and Kaback 2006; Law *et al.* 2008). The primary focus of these approaches, however, has been to target the residues involved in substrate binding and substrate access. An aspect that has lagged behind in these studies is the nature of the conformational changes in regions not directly involved in substrate access, and how these other global changes in conformation in the protein contribute toward the transport process.

Here, we have attempted to partially address this lacuna and describe an approach we used to perform “charged/polar residue scanning” of the hydrophobic face of a transmembrane helix of a membrane transporter; we followed this with detailed biochemical and genetic analysis. Hgt1p is a high-affinity glutathione transporter of the yeast *Saccharomyces cerevisiae* (Bourbouloux *et al.* 2000) that belongs to the relatively uncharacterized Oligopeptide Transporter family (Gomolplitinant and Saier 2011; Hauser *et al.* 2000; Lubkowitz 2011; Lubkowitz *et al.* 1997). Homologs in other yeasts have also been shown to function in glutathione transport (Bachhawat *et al.* 2013; Desai *et al.* 2011; Thakur and Bachhawat 2010; Thakur and Bachhawat 2013; Thakur *et al.* 2008) and in some cases oligopeptide transport (Osawa *et al.* 2006; Reuss and Morschhauser 2006), whereas more remote members are transporters of metal–amino acid conjugates (Curie *et al.* 2009; Curie *et al.* 2001; Inoue *et al.* 2009; Yen *et al.* 2001). The *S. cerevisiae* paralogue, OPT2, also appears to have a role in glutathione homeostasis while functioning at the yeast peroxisomes (Elbaz-Alon *et al.* 2014).Very little structure–function information is available for members of this family, and the lack of functionality of a cysteine-free mutant has made it difficult to apply methods, such as the Substituted Cysteine Accessibility Method (SCAM) (Frillingos *et al.* 1998; Kaur *et al.* 2009). The TMD9 of Hgt1p has been identified as being important in substrate binding, and two key residues, F523 and Q526, are thought to line the channel on the hydrophilic face of the helix (Kaur and Bachhawat 2009a; Thakur and Bachhawat 2010).On the basis of the helical wheel arrangement of TMD9, it appears that the hydrophobic side of the helix might interact with the lipid matrix and/or other transmembrane segments of the protein. Residues of this face of the TMD9 helix were systematically replaced with lysine, glutamine, and glutamic acid. These replacements in the hydrophobic patch are expected to be deleterious to the protein and would then be subjected to mutagenesis to identify functional suppressors in other parts of the protein, with the premise that critical interaction disrupted by the primary mutation would be compensated by mutations at second sites.

Among the different charged mutants created, only six were nonfunctional, revealing a surprising tolerance of charged residues in the hydrophobic part of TM helices. I524, proximal to the substrate binding residues, was the only position that did not tolerate any charged residues. Suppressor analysis of all the nonfunctional mutants yielded second-site suppressors only in the case of I524K and I524Q, both of which involved a G202Q, G202K, or G202I substitution in the hydrophilic loop of Hgt1p between TMD3 and TMD4, but G202Q/K/I alone was not deficient in transport activity. Charged and polar residue mutagenesis of P525, another residue close to the substrate binding residues, revealed that mere proximity to these residues was not responsible for the observations with I524. The results suggest that I524 in the hydrophobic face is in a conformationally critical region for substrate translocation and requires the involvement and possible interaction with region G202, near TMD3.

## Materials and Methods

### Chemicals and reagents

All the chemicals used in this study were analytical grade and obtained from commercial sources. Media components were purchased from Difco (Detroit, MI) Sigma Aldrich, (St. Louis, MO), HiMedia, (Mumbai, India), Merck India Ltd (Mumbai, India), and USB Corporation (Cleveland, OH). Oligonucleotides were purchased from Sigma India. Restriction enzymes, Vent DNA polymerase, and other DNA-modifying enzymes were obtained from New England Biolabs (Beverly, MA). DNA sequencing kit (ABI PRISM 310 XL with dye termination cycle sequencing ready reaction kit) was obtained from Perkin Elmer, (Norwalk, CT). Gel-extraction kits and plasmid miniprep columns were obtained from QIAGEN (Valencia, CA) or Sigma (St. Louis, MO). [^35^S] GSH (specific activity 1000 Ci mmol^-1^) was purchased from Bhabha Atomic Research Centre, Mumbai, India. HA-Tag (6E2) mouse monoclonal antibody and horse anti-mouse HRP-linked antibody were bought from Cell Signaling (Danvers, MA). Alexa Flour 488 conjugated goat anti-mouse antibody was obtained from Molecular Probes (Eugene, OR). Hybond ECL (nitrocellulose) membrane and ECL plus Western blotting detection reagents were purchased from Amersham Biosciences (UK).

### Strains, media, and growth conditions

The *Escherichia coli* strain DH5α was used as a cloning host. The *Saccharomyces cerevisiae* strain used in this study was ABC 817 *(MATa his3Δ1 leu2Δ0 met15Δ*-0 *ura3Δ0 hgt1Δ*::*LEU2)* (Bourbouloux *et al.* 2000). *S. cerevisiae* was regularly maintained on yeast extract, peptone, and dextrose (YPD) medium. *S. cerevisiae* synthetic defined minimal medium (SD) contained yeast nitrogen base, ammonium sulfate, and dextrose supplemented with histidine, leucine, and methionine (when required) at 50 mg/liter (Guthrie and Fink 1991). Glutathione was added as required. Growth, handling of bacteria and yeast, and all the molecular techniques used in the study were according to standard protocols (Sambrook *et al.* 1989).

### Site-directed mutagenesis

*HGT1*, tagged with a Hemagglutinin (HA) tag at the C-terminus, was subcloned downstream of the TEF promoter at the *Bam* HI and *Eco* RI sites of a modified p416TEF vector (Kaur *et al.* 2009). This construct was used as a template for site-directed mutagenesis for creation of different site-directed mutants of Hgt1p by splice overlap extension strategy. The mutations, K,Q,E, for each residue were generated using a single mutagenic oligonucleotide exploiting degenerate base pairs at the desired position in the different mutagenic oligonucleotides (Supporting Information, Table S1). The PCR products generated with these oligonucleotides were subcloned back into the TEF vector background using appropriate restriction sites for subsequent analyses. The resulting mutants were sequenced to confirm the presence of the desired nucleotides changes and to rule out any undesired mutations introduced during the mutagenic procedure.

### The dual complementation-cum-toxicity plate assay for assessing *HGT1* functionality

The complementation-cum-toxicity assay has been described previously (Kaur *et al.* 2009). The *S. cerevisiae met15*Δ *hgt1*Δ (ABC 817) was transformed with a single-copy centromeric vector expressing wild-type or different mutants of TMD9 of *HGT1* expressed downstream of the TEF promoter. Transformants were grown in minimal media containing methionine and other supplements, without uracil, overnight. These cultures were reinoculated in the same media and allowed to grow until they reached the exponential phase. An equal number of cells were harvested, washed with water, and resuspended in sterile water to an OD600 of 0.2. These were serially diluted 1:10, 1:100, and 1:1000; 10 μl of these cell resuspensions were spotted on minimal medium containing different concentrations of glutathione (15, 30, 50, 100, 150, 200 μM) or methionine (200 μM) as sole organic sulfur source. Because *HGT1* is under the strong TEF promoter, it is able to confer the ability to grow on glutathione at low concentrations of glutathione. However, at higher concentrations of glutathione, excessive uptake leads to toxicity in growth. Using this growth at low concentrations, and lack of it at higher concentrations, we have been able to grade the transporter defects, which is more informative than a mere growth assay at lower concentrations. The plates were incubated at 30° for 2–3 d and photographs were taken.

#### Hydroxylamine mutagenesis of the pTEF-HGT1:

The random *in vitro* mutagenesis of the charged/polar residues of *HGT1* was done using hydroxylamine and a previously described protocol (Rose and Fink 1987; Thakur and Bachhawat 2013). The 10 μg plasmid DNA was incubated in 0.5 ml of hydroxylamine solution (90 mg NaOH, 350 mg hydroxylamine HCl in 5 ml water, pH ∼6.5, freshly made before use). This mixture was incubated at 37° for 24 hr and mutagenized DNA was directly purified using Qiagen column. The purified plasmid DNA concentration was measured by running on agrose gel along with DNA ladder. The randomly mutagenized plasmid was directly transformed in *S. cerevisiae met15Δ hgt1Δ* strain and selected on 10 µg glutathione containing plates, and functional transformants were used to prepare plasmid DNA following a retransformation.

### Glutathione transport assay

The *S. cerevisiae* ABC 817 strain (*met15Δhgt1Δ*) was transformed with different plasmid constructs bearing wild-type or *HGT1* mutants under TEF promoter and was grown in minimal media containing methionine and other supplements, without uracil, overnight. These cultures were reinoculated in the same media and allowed to grow until they reached the exponential phase. Cells were harvested, washed, and put on ice in a MES-buffered medium until the transport was initiated. Transport experiments were carried out with [35^S^]-GSH as described earlier (Kaur *et al.* 2009). The results were expressed as nmol of glutathione-mg.protein-1 min-1. For the measurements of total protein, the 100 μl of the above cell suspension (cell suspension volume used for the transport assay) was boiled with 15% sodium hydroxide for 10 min, followed by neutralization of total cell lysate by addition of hydrochloric acid; 100 μl of this crude cell lysate was incubated with 0.1% Triton X-100 for 10 min and total protein was estimated by using the Bradford reagent (Sigma) using bovine serum albumin as a standard. For saturation kinetics, the initial rate of glutathione uptake was measured at a range of glutathione concentrations from 12.5 μM to 800 μM, with specific activity being kept constant at each concentration. The initial rate of glutathione uptake was determined by measuring the radioactive glutathione accumulated in the cells at 30-sec and 180-sec time points in the ABC 817 strain transformed with different plasmids constructs bearing wild-type and *HGT1* mutants under TEF promoter or only vector. After subtracting the initial rate of glutathione uptake in vector from the initial rate of glutathione uptake in the different test constructs, the extent of glutathione uptake corresponding to the test construct was determined.

### Preparation of cell extract and Western blot analysis

Crude cell extracts were prepared and Western blot analysis was done as described previously (Kaur *et al.* 2009) using a modified Western blot method (Kaur and Bachhawat 2009b). Densitometry analysis of the unsaturated band signals was performed using the Scion Image software to quantify the protein expression levels in the different mutants. The resulting signal intensity was normalized with respect to the band surface area (in square pixels) and expressed in arbitrary units. The relative protein expression levels in the mutant Hgt1p were represented as percentage expression relative to wild-type Hgt1p.

### Cellular localization of the mutants by confocal microscopy

To localize the different charged residue mutants of TMD9 of Hgt1p that were created, indirect immunofluorescence was performed using a published protocol modified as described earlier (Kaur *et al.* 2009). For staining of ER (endoplasmic reticulum), live yeast cells were incubated with the ER-Tracker TM Red dye (BODIPY TR glibenclamide; Invitrogen, USA) according to the manufacturer’s instructions and by other published literatures (Feng *et al.* 2010; Mandal *et al.* 2012; Rawal *et al.* 2013). Images were obtained with an inverted LSM510 META laser scanning confocal microscope (Carl Zeiss) fitted with a Plan-Apochromat ×100 (numerical aperture, 1.4) oil immersion objective. The 488-nm line of an argon ion laser was directed over an HFT UV/488 beam splitter, and fluorescence was detected using an NFT 490 beam splitter in combination with a BP 505 to 530 band pass filter. ER-Tracker fluorescence was detected at Ex/Em 587/615. Images obtained were processed using Adobe Photoshop version 5.5

## Results

### Analysis of the residues of the hydrophobic face of the TMD9 helix by mutation to charged residues

**Figure 1:**
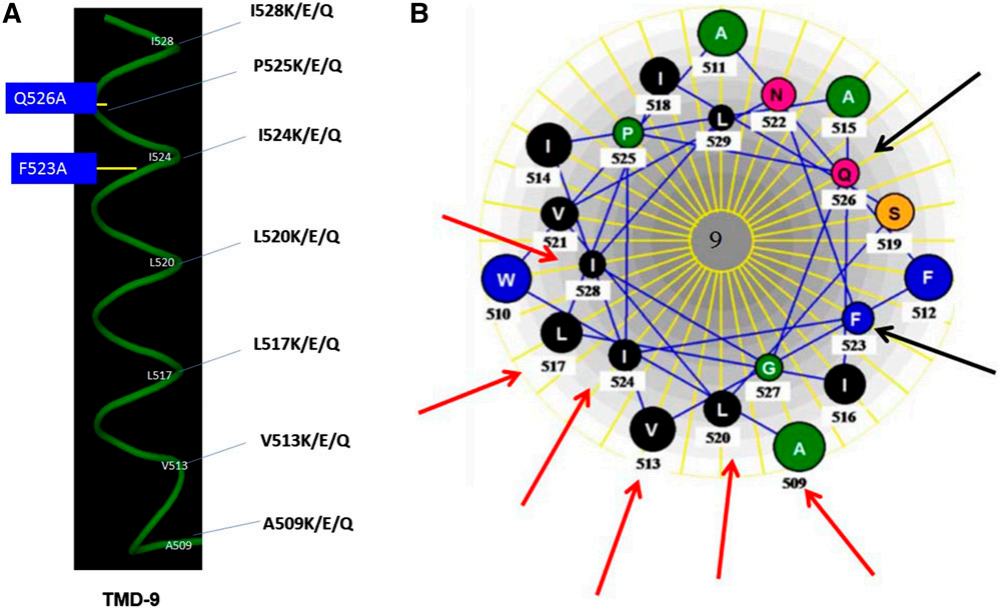
Residues of TMD9 selected for charged residue scanning. (A) The side view of the helix (shown in green). The residues selected for charged residue scanning indicated by their position. The side view of the TMDs was drawn using the PyMol Molecular viewer (version 0.99). The blue boxes represent residues involved in substrate binding. (B) Helical wheel representation of helix 9 of Hgt1p viewed from the exoplasmic surface of the membrane. Amino acid representation by the single letter code. The red arrow points to the residues selected for charged residue scanning situated on the hydrophobic face of the respective transmembrane domains. Helical wheel model of the transmembrane helix 9 of Hgt1p was constructed using the Lasergene software (DNAstar, Madison, WI).

The helical wheel diagram of TMD9 shows a topological sidedness. The residues F523 and Q526, shown to be important in substrate binding, are located on the more polar face of the channel and are thought to directly interact with the substrate (Kaur and Bachhawat 2009a; Thakur and Bachhawat 2010). The hydrophobic face of the helix, in contrast, is expected to interact with the lipid bilayer or with other TMDs. These residues in TMD9 are A509, V513, L517, L520, I524, and I528. To investigate this face and these residues more systematically, we considered a novel strategy. In this approach, each of the residues on this face was mutated to multiple charged residues, lysine, glutamine, or glutamic acid (using a single degenerate oligonucleotide), and nonfunctional mutants would then be subjected to suppressor analysis to obtain further insights (Figure 1, A and B). The residues described above were mutated as indicated and the mutants were subjected to an initial functional characterization using the previously designed sensitive plate assay, termed a complementation-cum-toxicity assay (Kaur and Bachhawat 2009a; Kaur *et al.* 2009). The assay is based on the ability of *HGT1* when expressed from the TEF promoter to permit growth on glutathione as a sole sulfur source in an *S. cerevisiae* strain (ABC 817). This strain is an organic sulfur auxotroph defective in glutathione uptake. At low glutathione concentrations (15 µM), *HGT1* expressed under the TEF promoter complements the growth defect in the ABC 817 strain (Bourbouloux *et al.* 2000) but results in toxicity when cells are grown in medium containing 50-µM or higher glutathione concentrations (Kaur *et al.* 2009; Srikanth *et al.* 2005). The reasons for this toxicity have been examined elsewhere (Kumar *et al.* 2011), but the dual assay permits one to discern a gradation in functionality. Plasmids bearing either the WT *HGT1* or the different mutants as described above were individually transformed into the *S. cerevisiae* ABC 817 strain, and the transformants were analyzed for their ability to confer complementation and/or toxicity to the cells over a range of glutathione concentrations.

**Figure 2:**
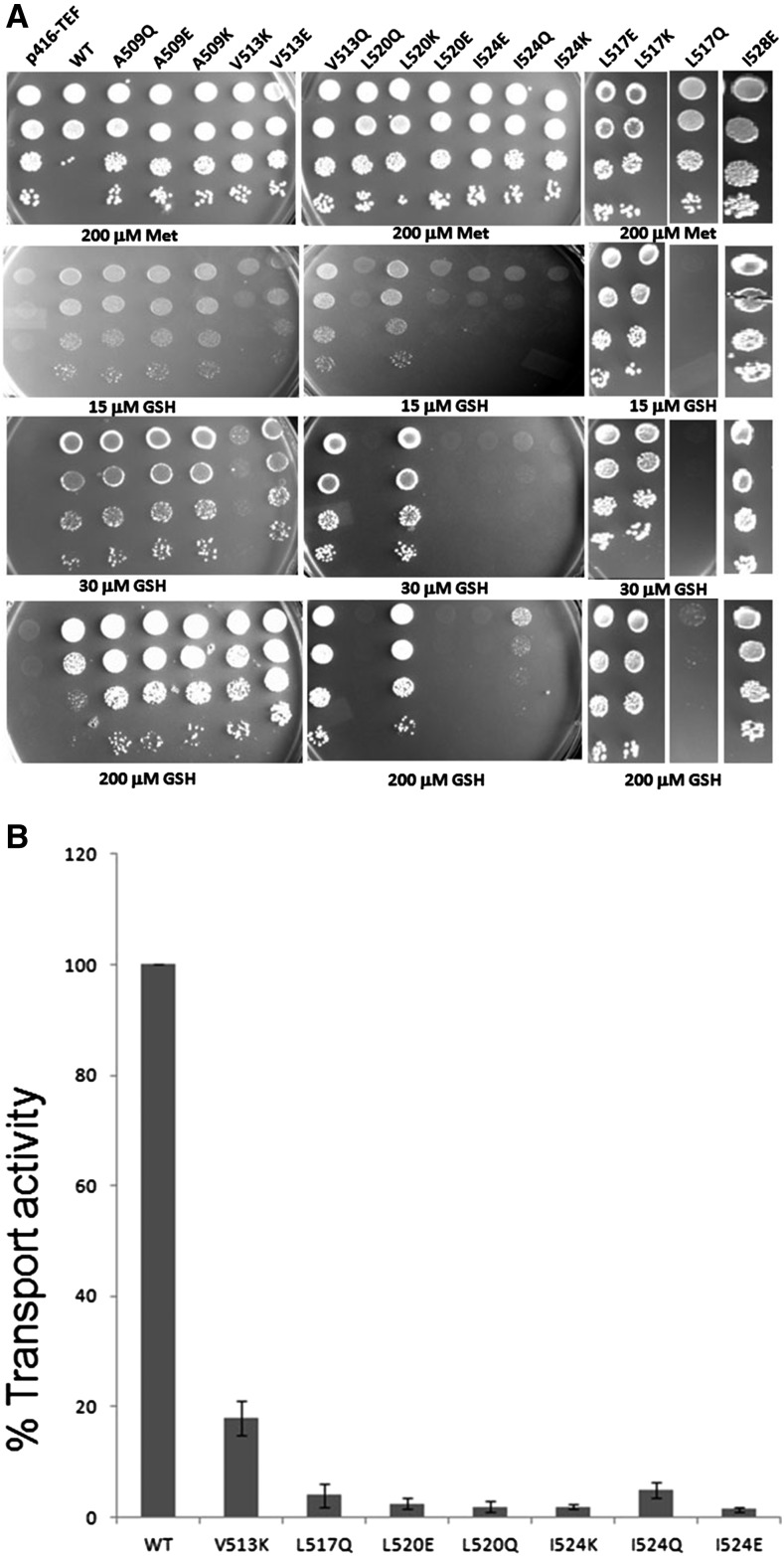
(A) Growth pattern of charged/polar residue mutants of transmembrane domain 9 of Hgt1p when grown on different concentrations of glutathione. Hgt1p and the different charged/polar mutants of TMD9 of Hgt1p expressed under the TEF promoter, and corresponding vector (p416TEF) were transformed into the *S. cerevisiae* strain ABC817 and evaluated by the complementation-cum-toxicity assay by dilution spotting on minimal media containing glutathione. Transformants were grown in minimal medium containing methionine, harvested, washed, and resuspended in water and serially diluted to give 0.2, 0.02, 0.002, and 0.0002 OD_600_ of cells; 10 μl of these dilutions was spotted on minimal medium containing different concentrations of glutathione (GSH). The photographs were taken after 3 d of incubation at 30°. (B) Relative rate of glutathione uptake in selected mutants of TMD9 mutated to charged residues. Hgt1p and the different mutants of TMD9 of Hgt1p expressed under the TEF promoter were transformed into strain *met15Δ hgt1Δ* and percentage transport activity was determined relative to wild-type Hgt1p as described in the experimental procedures.

Based on their inability to complement the glutathione transporter defect, the mutants L517Q, L520Q, L520E, I524E, I524K, and I524Q were nonfunctional. V513K was severely defective but still mildly functional. The remaining mutants (A509Q, A509K, A509E, V513Q, V513E, L517K, L517E, L520K, and I528E) surprisingly had no functional defect and were comparable to WT (Figure 2A and Table 1). In case of I528, only I528E was constructed, because it was the only mutant plasmid recovered among the 20 different mutants of I528 that we analyzed.

**Table 1:** Summary of the charged residues mutants of *HGT1*

Residue	Mutants	Functional Activity
A509	A509Q	+++
	A509K	+++
	A509E	+++
V513	V513Q	+++
	V513K	+
	V513E	+++
L517	L517Q	—
	L517K	+++
	L517E	+++
L520	L520Q	—
	L520K	+++
	L520E	—
I524	I524Q	—
	I524K	—
	I524E	—
I528	I528E	+++

Summary of the charged residue mutants of *HGT1* on functional activity of the transporter using dual complementation-cum-toxicity assay.

To corroborate these growth assays with biochemical assays, the mutants that were nonfunctional and severely defective were evaluated by measuring the rate of ^35^S-GSH uptake in the ABC 817 strain. The uptake data correlated well with the plate assay. Among the defective mutants, V513Q displayed some transport activity (∼18%) in agreement with the plate assay. However, the others mutants L517Q, L520Q, L520E, I524E, I524Q, and I524K had an almost complete loss in functional activity, showing very minimal uptake ability in agreement with the plate assay (Figure 2B)

### Expression and cell surface targeting of nonfunctional mutants

**Figure 3:**
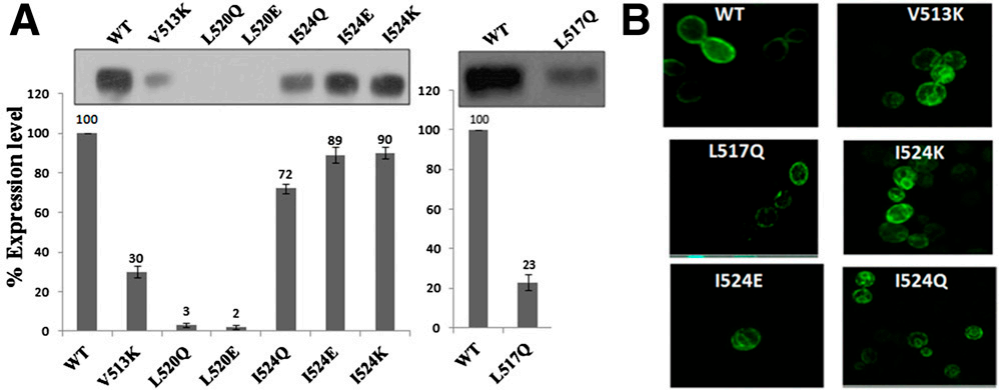
(A) Quantification of the total protein expression levels of the different mutants of Hgt1p. Protein extracts were prepared from the *S. cerevisiae* strain ABC817 transformed with plasmid bearing either the WT or the different charged/polar residues mutants of TMD9 of Hgt1p; 20 μg of total protein was loaded onto SDS-PAGE, resolved, and electroblotted to a nitrocellulose membrane. The data are expressed as percentage protein expression normalized to the wild-type expression level and is mean of the protein expression levels obtained in two independent experiments. A representative blot is shown in the inset. Equal loading of the proteins (20 μg) in each well of the gel was also visually monitored by Coomassie staining and Ponceau S staining of the membrane after transfer (data not shown). (B) Cell surface localization of charged/polar residues mutants of Hgt1p lacking functional activity. The *S. cerevisiae* strain ABC817 was transformed with plasmids bearing either the WT or the different charged/polar residues mutants of TMD9 of Hgt1p and labeled by indirect immunofluorescence using HA monoclonal antibody as a primary antibody, followed by the Alexa 488, and visualized using confocal microscopy, as described in experimental procedure. Only fluorescence images have been shown.

To determine whether the nonfunctional mutant proteins are expressed and targeted properly, we first examined their steady-state expression levels by immunoblotting. Equal amounts of the crude protein extracts prepared from the *met15∆hgt1∆* strain transformed with the different mutants were loaded onto the gel, electroblotted to the membrane, and probed with anti-HA monoclonal antibody. L520Q and L520E showed almost complete loss in protein expression level. V513K and L517Q mutant showed detectable, but significantly lower, expression, whereas the other mutants I524K, I524Q, and I524E showed slightly lower, but otherwise comparable, levels to WT, and their expression ranged between 65% and 92% of wild-type (Figure 3A).

The nonfunctional mutants were also studied for subcellular localization by indirect immunofluorescence using anti-HA monoclonal antibody. No signal was observed either in L520Q or in L520E, which probably reflects the almost complete loss of protein expression. In V513K, L517Q, I524Q, I524E, and I524K, a signal was observed at the cellular periphery of the cells, although they also showed a small amount of intracellular signal in addition to the cell surface signal (Figure 3B, Figure S1). This suggested that these mutants did not carry a significant trafficking defect.

### G202K/I and G202K/Q mutations in the loop region between TMD3 and TMD4 are able to restore function to the nonfunctional I524K and I524Q mutants

Among all the hydrophobic residues that were evaluated by this charged residue scanning, it was only the charged residues at position I524 (I524K, I524K, and I524Q) that could not be tolerated as seen in functional assays. At all other positions, at least one of the charged residues was tolerated (Table 1).

The loss of function mutants I524K/Q/E were further subjected to suppressor analysis to examine if these mutants that were otherwise properly expressed and localized might be restored to functionality by compensatory mutations. Mutants were mutagenized *in vitro* with hydroxylamine treatment and transformed into the yeast ABC 817 strain. The resulting transformants were screened for a functional phenotype by direct selection on minimal media containing 10 μM GSH. For those clones bearing the original mutation, the full gene was sequenced to identify any additional mutations. The I524Q mutation yielded eight suppressors, of which two were revertants, whereas the remaining were either G202K or G202Q (Table 2). The I524K mutations yielded 10 suppressors, of which five were revertants, whereas the remaining were either G202K or G202I (Table 2). It is interesting that in all cases it was the G202 that was mutated to different amino acids (G202Q, G202K, and G202I) (Table 2). G202 is located in the intracellular loop between TMD3 and TMD4, but at the junction of TMD3.

**Table 2:** Suppressor isolated from hydroxylamine mutagenesis–based intragenic suppressor analysis of mutant*s*

Primary Mutation	No. of Suppressors Obtained at 10 µM of GSH Concentration	Suppressor Distribution (No.)
V513K	6	513V (4)
		513N (1)
		513Q (1)
L517Q	4	517L (3)
		517A (1)
L520Q	6	520L (6)
L520E	8	520L (8)
I524Q	8	524I (2)
		I524Q G202Q (2)
		I524Q G202K (4)
I524E	8	524I (8)
I524K	10	524I (5)
		I524K G202K (3)
		I524K G202I (2)

We also subjected the remaining nonfunctional mutants to this suppressor analysis. The V513K suppressor involved same-site mutations with conversion to 513N and 513Q. Similarly, the L517Q suppressors yielded a mutation at the same site, 517A. In the case of L520Q and L520E, several suppressors were isolated but they all reverted back to the wild-type sequence. Thus, second-site suppressors were obtained only for I524Q and I524K mutations and were found to involve the G202 residue.

To investigate the I524-G202 interaction in greater detail, we created several additional charged mutants of I524 (I524D, I524R, I524N) as well as G202 (G202D, G202R, G202N, G202E) and evaluated the functional interaction of the different combinations (Table 3). Interestingly, the fresh charged residues at the I524 position that we introduced were all nonfunctional, although some partial functionality was observed with the I524N mutant.

**Table 3:** Genetic analysis of I524 and G202 mutants and interaction involving I524-G202 in functional activity of the Hgt1p using dual complementation-cum-toxicity assay

Residue at position 524	Residue at Position 202	Functional Activity
I	G	+++ (WT)
Q	G	—
E	G	—
K	G	—
D	G	—
R	G	—
N	G	−/+
I	K	+++
I	I	+++
I	Q	+++
Q	K	+++
Q	Q	+++
Q	R	+++
Q	D	—
Q	N	—
Q	E	—
K	I	+++
K	K	+++
K	N	—
K	D	—
K	E	—
R	R	—
R	N	—
R	K	—
D	K	—
D	N	—
N	R	—

Detailed genetic analysis of I524 and G202 mutants and interaction involving I524-G202 in functional activity of the Hgt1p using dual complementation-cum-toxicity assay.

The interaction of these various combinations as measured by the growth assay is summarized in Table 3. Among the various G202 mutations that were made, only G202R and G202Q were able to suppress the I524Q mutation in a manner similar to G202K and suggested that the charge or polarity at this position was critical for suppression. However, in contrast, the I524R mutant (unlike the I524K mutant) could not be suppressed by G202K. Disruption of the activity by this mutation was therefore causing other defects in the functionality. None of the other mutations that included negatively charged residues could restore functionality in these interactions.

### Functional and kinetic analysis of the I524K and I524Q suppressors

**Figure 4:**
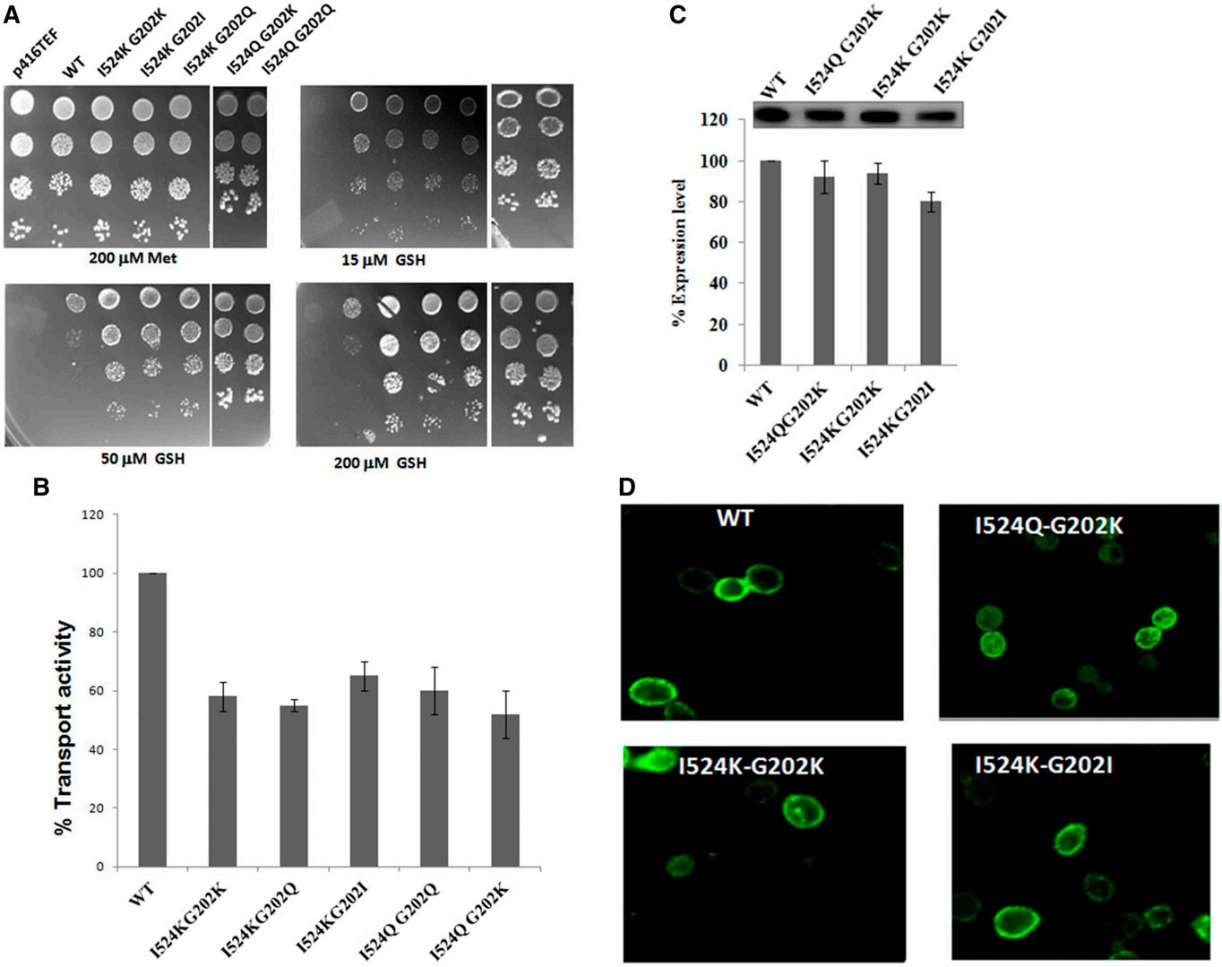
(A) Growth pattern of I524Q and I524K suppressors. Plasmids bearing the I524KG202K, I524KG202I, I524KG202Q, I524QG202K, and I524QG202Q and the corresponding WT or vector (p416TEF) were transformed into *S. cerevisiae* strain ABC817 and evaluated by the complementation-cum-toxicity assay by dilution spotting on minimal media containing glutathione. (B) Relative rate of radiolabeled glutathione uptake of I524Q and I524K suppressors. Plasmids bearing the I524K G202K, I524K G202Q and I524K G202I, I524Q G202Q and I524Q G202K, and the corresponding WT or vector (p416TEF) were transformed into *S. cerevisiae* strain ABC817 and transport rates were determined as described in experimental procedures. The data are represented as percentage rate of glutathione uptake by the suppressors relative to wild-type Hgt1p (WT). (C) Quantification of the total protein expression levels of suppressors of I524Q and I524K. Extracts were prepared from the *S. cerevisiae* strain ABC817 transformed with plasmids bearing either the WT or the I524Q G202K, I524K G202K, and I524K G202I suppressors, resolved on SDS-PAGE, and electroblotted onto a nitrocellulose membrane. The protein bands were quantified by densitometry scanning. The data are expressed as percentage protein expression normalized to the wild-type expression level and are the mean of the protein expression levels obtained in two independent experiments. A representative blot is also shown. Equal loading of the proteins (20 μg) in each well of the gel was also visually monitored by Coomassie staining and Ponceau S staining of the membrane after transfer (data not shown). (D) Cell surface localization of I524Q and I524K suppressors. The *S. cerevisiae* strain ABC817 was transformed with plasmids bearing the I524K G202K, I524K G202I, and I524QG202K suppressors of TMD9 of Hgt1p and labeled by indirect immunofluorescence visualized using confocal microscope. Only fluorescence images are shown.

To evaluate the suppressors in greater detail, the mutants I524K G202K, I524K G202I, I524K G202Q, I524Q G202K, and I524Q G202Q were spotted on different concentrations of glutathione and evaluated by the dual complementation-cum-toxicity assay. The suppressors G202K, G202Q, and G202I with their primary mutation I524K and I524Q were able to suppress the phenotypic defect caused by I524Q and I524K in terms of their ability to complement growth on glutathione. All these suppressors exhibited a functional activity almost similar to wild-type in being able to complement at lower concentrations of glutathione. However, the suppressors displayed a slight loss in functional activity in terms of their ability to confer toxicity to the cells at high concentrations of glutathione (Figure 4A). To corroborate the growth assays with actual transport data, we measured the initial rate of ^35^S-glutathione uptake in the ABC 817 strain transformed with the suppressor mutants of Hgt1p. The uptake was 45–62% as compared to wild-type (Figure 4B). These results indicated that the suppressors had regained significant activity.

To determine the effect of suppressor mutations in the absence of the primary mutation, the G202Q, G202K, and G202I mutations were subcloned into the wild-type plasmid backbone. The mutants were functionally evaluated using the plate based dual complementation-cum-toxicity assay (Figure S2). The individual mutants G202Q G202K and G202I appeared to have an activity similar to wild-type. The protein expression levels of these suppressors with primary mutation were also similar to wild-type and properly localized to the cell surface (Figure 4, C and D).

Because of the low activity of the I524K/E/Q mutations, their kinetics could not be evaluated. However, the restoration of activity by the G202K/I/Q suppressors in these backgrounds provided the opportunity to get some insight into these mutants. We determined the kinetic parameter by measuring the initial rate of glutathione uptake over a range of glutathione concentrations. I524K and I524Q had almost null activity and could not be included for the kinetic study. We determined the kinetic parameters for I524Q G202K. Compared to the *Km* of the WT 48.1 ± 10.5 μM (Bourbouloux *et al.* 2000), the *Km* for the suppressor mutant was significantly higher at 282.8± 44.5 μM (Figure S3).

### Charged/polar residue scanning of P525 of TMD9 of Hgt1p

**Figure 5:**
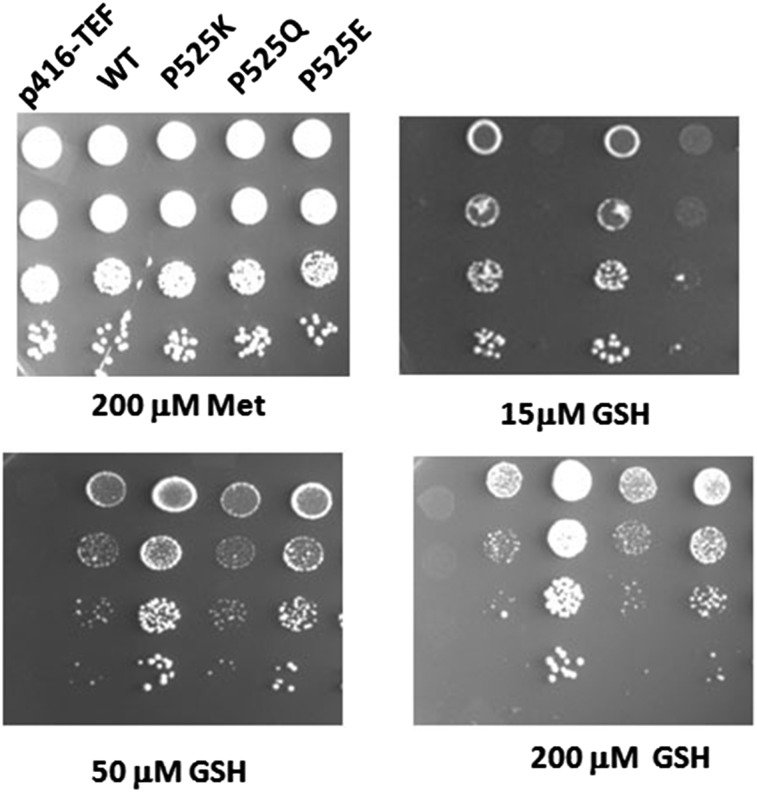
Functional characterization of P525K, P525Q, and P525E mutants of Hgt1p. The P525K, P525Q, and P525E mutants expressed under the TEF promoter, the WT, and corresponding vector (p416TEF) were transformed into *S. cerevisiae* strain ABC817 and evaluated by the complementation-cum-toxicity assay by dilution spotting on minimal media containing different concentrations of glutathione.

As I524 was located between the previously identified substrate binding residues F523 and Q526, examination was needed regarding the possibility that the inability of I524 to tolerate charged/polar residues might be due to this proximity to the substrate binding site rather than as a consequence of it being on the hydrophobic face and being involved in interactions critical for the channel functioning. To evaluate this possibility, we decided to examine if the residue, P525 adjacent to I524 and also located between F523 and Q526, could tolerate any charged/polar residues. P525 was mutated to lysine, glutamine, and glutamic acid by site-directed mutagenesis. These mutants were also subjected to an initial functional characterization by growth assays. P525K and P525E showed a severe effect on functional activity of Hgt1p, although they still retained partial activity, but P525Q had no discernible loss in function and was comparable to WT (Figure 5). Thus, polar residues at this position could be tolerated despite the proximity to F523 and Q526.

The severely affected mutants P525K and P525E were also subjected to suppressor analysis. Suppressors were screened for a functional phenotype by direct selection on a minimal media containing 10 μM GSH. Plasmids were retrieved, re-examined for their functional activity using the growth-based plate assay, and sequenced. In one clone, P525K had converted to 525Q. All other suppressors for both P525K and P525E had reverted back to the wild-type sequence P525.

## Discussion

The work described in this manuscript has explored a novel strategy for gaining insight into the mechanisms of membrane transporter functioning. In this approach, the six residues that formed the hydrophobic face of a TMD helix whose polar face was presumed to line the substrate translocation channel was subjected to “charged/polar-residue scanning” mutagenesis followed by genetic suppressor and biochemical analysis.

One of the most striking observations revealed from this study was that membrane transporters are surprisingly tolerant of charged residues, even in the most hydrophobic part of the TMDs. Previous efforts whereby charges were introduced into TM domains of membrane proteins were done either to examine the role in translocation/trafficking (Davis and Hunter 1987) or to determine if the membrane domain spanned the membrane lipid bilayer with a helical (Song *et al.* 1991). In both cases, introduction of charged residues led to significant loss in function of the proteins. In contrast, out of the 16 mutations made to charged/polar residues (to lysine, glutamine, or glutamic acid), only six of these showed a complete loss in activity. In those cases, when an effect was seen, the effect of the replacement depended on both the position and the residue being introduced. Thus, for example, V513Q was functional, but V513K showed very low functionality; in contrast, L517Q was nonfunctional but L517K was functional. The loss of functionality in these cases was shown to be due to loss of protein expression, but it is clear that in the absence of any structural information it is difficult to predict the effects of introducing different charge/polarity at a particular position. Only at one of the six positions, position I524, was there a complete lack of tolerance to any charged/polar residues. I524E, I524K, or I524Q were all completely nonfunctional. However, all other positions on this face were capable of accepting at least one of the charged/polar residues. The I524 residue mutated to charged residues was also the only residue that allowed isolation of intragenic suppressors. This might suggest that other residues are not directly involved in interaction with other domains, and might also be an explanation for why charged/polar residues at these positions are more easily tolerated

Among the six substituted changes that led to complete loss in activity, three of them, L520Q, L520E, and L517Q, appeared to have destabilized the protein because no protein could be detected in L520Q and L520E and a very reduced protein expression was seen in L517Q. It was interesting that charged/polar residue changes at I524 affected the function of the protein but did not affect the protein expression to any significant extent. These mutants were also properly localized to the cell surface. There are thus two possible explanations for the complete loss in activity seen in I524K, I524Q, and I524E. The first one is that considering the proximity to the substrate binding residues; the changes were somehow drastically interfering with the substrate-binding. This could have been resolved by kinetic analysis; however, the activity was too low to allow us to subject these mutants to any kind of kinetic analysis. We thus examined how P525, another residue located between F523 and Q526, could tolerate charged residues. We subjected P525 to charged/polar residue scanning mutagenesis. P525K and P525E were nonfunctional, but P525Q was functional. Thus, charged/polar residues were functional at position P525, suggesting that mere proximity to the substrate binding residues could not account for the behavior of the I524K/Q/E mutants.

That brings us to the second plausible explanation for the complete loss of function of the I524K, I524E and I524Q mutants, namely, that the residue was on the region of the hydrophobic face that was part of the dynamics of channel movement and function. If this was the case, then it should involve interactions with other domains and, if so, we should be able to isolate suppressor mutations at other parts of the protein that might be able to suppress this loss of function. It is interesting in this context that although all the nonfunctional mutants were subjected to suppressor analysis, only second-site suppressor mutations were identified for I524Q and I524K. The position at which the second-site mutations had occurred was at G202, and it is predicted to be located in the loop between TMD3 and TMD4, close to TMD3. Interestingly, neither of these residues (neither I524 nor G202) was conserved in the oligopeptide transporter (OPT) super family. Interestingly, second-site suppressors were not observed even in the search for suppressors of P525K and P525E.

G202Q by itself is not an inhibitory mutation, but in the background of I524Q and I524K it functioned as a genetic suppressor that reverses the incapacitating constraints introduced by the I524Q and I524K mutation. Interestingly, the second-site suppressor was always found at the G202 position for both nonfunctional mutations I524Q and I524K. These studies suggest that TMD9 and TMD3 may be adjacent to, or in close contact with, each other. Sequence analysis of OPT homologs for any evolutionary coupling of G202 and I524 was carried out; however, no such coupling seemed to exist (data not shown). The higher *K_m_* in the suppressor suggests that the interaction has an impact on the substrate binding, but the major role of this interaction seemed to go beyond the effects on substrate binding. An explanation for the G202 with Q/K/I substitutions is that it could shift the local secondary structure probability from loop to helix and may produce helical turn in TMD3. Irrespective of this or other effects of G202 mutations, it is clear that the region plays a critical role in substrate translocation in conjunction with the hydrophobic face of TMD9. More biophysical, biochemical, or crystal structure studies would be required, however, to confirm these events.

In conclusion, we have used a novel strategy that we refer to as charged/polar-residue scanning mutagenesis of the hydrophobic face of TM helices as an approach to investigating membrane transporters and that could be added to the list of approaches currently being used to investigate this class of proteins. Genetic suppressor analysis for the determination of interacting domains is not a new approach, even in membrane transporters, and has been successfully applied previously (Arastu-Kapur *et al.* 2005; Auerbach *et al.* 2002), but the systematic application of this strategy to the hydrophobic face of the helix reveals a novel way of gaining insight into this class of proteins. The application of this approach to TMD9 of the yeast glutathione transporter, Hgt1p, has yielded several important insights, and we believe that this approach can be successfully applied to other transporters as well.
